# Autophagy-Based Unconventional Secretory for AIM2 Inflammasome Drives DNA Damage Resistance During Intervertebral Disc Degeneration

**DOI:** 10.3389/fcell.2021.672847

**Published:** 2021-06-22

**Authors:** Shuai Li, Zhiwei Liao, Rongjin Luo, Yu Song, Kun Wang, Xiaobo Feng, Yangliu Ou, Xinghuo Wu, Yukun Zhang, Yong Gao, Huipeng Yin, Cao Yang

**Affiliations:** Department of Orthopaedics, Union Hospital, Tongji Medical College, Huazhong University of Science and Technology, Wuhan, China

**Keywords:** absent in melanoma 2 inflammasome, DNA damage, intervertebral disc degeneration, autophagy, unconventional secretion

## Abstract

Intervertebral disc degeneration (IDD) is the primary cause of low back pain. Stress-induced DNA damage is closely relevant to the pathogenesis of IDD; however, the underlying mechanisms remain unclear. This study investigated the role of the absent in melanoma 2 (AIM2) inflammasome as a DNA damage sensor in nucleus pulposus (NP) cells. We found that the level of AIM2 increased in degenerated discs and was correlated to the degree of IDD. Knockdown of AIM2 ameliorated H_2_O_2_-induced DNA damage and apoptosis in NP cells *in vitro*, and retarded the progression of IDD *in vivo*. Furthermore, the induction of autophagy protected against cellular DNA damage via the unconventional secretion of AIM2. We further identified the Golgi re-assembly and stacking protein 55 (GRASP55) as mediator of the transport and secretion of AIM2 via an autophagic pathway. Taken together, our researches illustrate the role and regulatory mechanism of the AIM2 inflammasome during IDD. Targeting the AIM2 inflammasome may offer a promising therapeutic strategy for patients with IDD.

## Introduction

Low back pain (LBP) is the leading cause of chronic disability and contributes greatly to the global health burden (Dowdell et al., 2017). Intervertebral disc degeneration (IDD) characterized by diminishing resident cells and progressive loss of the extracellular matrix (ECM) is a critical risk factor for LBP (Vergroesen et al., 2015). The nucleus pulposus (NP) is located in central disc and is surrounded by an annulus fibrosus composed of collagen lamellae. NP cells produce and maintain diverse ECM components that provide the swelling properties and pressure buffering function of the disc (Risbud and Shapiro, 2014). It is commonly considered that injury and death of NP cells contributes significantly to the pathogenesis of IDD (Lan et al., 2020). However, the pathogenesis of NP cell damage is multifactorial, and the underlying mechanisms of IDD remain unclear.

DNA damage, including nuclear and mitochondrial DNA damage, can lead to cellular dysfunction and result in cell death or senescence, which tightly links to degenerative diseases (Madabhushi et al., 2014). The occurrence and progression of IDD are closely associated with oxidative stress and DNA damage (Nasto et al., 2014). The absent in melanoma 2 (AIM2) inflammasome is a recognized cellular DNA sensor that serves as the monitor and reactor of cellular DNA damage (Lugrin and Martinon, 2018). AIM2 activation initiates the assembly of inflammasome complex and leads to the inflammatory cascades, which induce the release of inflammatory cytokines (Kumari et al., 2020). It has been reported that inflammatory stimuli and activated inflammatory responses facilitate the progression of IDD; however, the role of AIM2 inflammasome activation in IDD is still unknown.

Autophagy is a conserved cellular process responsible for the degradation and recycling of damaged organelles and proteins (Mizushima and Komatsu, 2011). Moderately active autophagy is cytoprotective and protects against IDD progression, and the inhibition of autophagy promotes the degradation of ECM components and cell apoptosis (Zhang et al., 2016). During the progression of IDD, autophagy may play a protective role via regulating the level of oxidative stress and the activation of inflammasomes (Feng et al., 2017). In a typical autophagy process, fusion of autophagosome with lysosome facilitates the degradation of autophagic cargoes (Ponpuak et al., 2015). In addition to regular degradation in autolysosomes, autophagic cargoes also rely on the autophagy-dependent secretion to eliminate harmful proteins or aggregates (Ponpuak et al., 2015). Autophagy-dependent secretion can release a plethora of factors which lack a secretion signal sequence, including interleukins, damage response mediators, and some extracellular matrix components (New and Thomas, 2019). Several studies have reported the extracellular distribution of the AIM2 inflammasome, which contributes greatly to the development of diseases (He et al., 2020; Lammert et al., 2020; Yuan et al., 2020). It is unclear if the secretion of the AIM2 inflammasome is autophagy-dependent.

In this study, we investigated the role of the AIM2 inflammasome in IDD progression. We measured the expression of the AIM2 inflammasome in non-degenerative and degenerative disc tissues. The activation of AIM2 inflammasome contributed to the release of inflammatory cytokines and cell death in the H_2_O_2_-induced DNA damage model of NP cells. On the other hand, activation of autophagy exerted a protective effect on DNA damage and apoptosis in NP cells, which partly depends on the secretory of AIM2. AIM2 colocalized with autophagic marker, LC3, and release to extracellular region upon the autophagy activation. In the rat disc IDD model, we determined the role of AIM2 during the progression of IDD *in vivo*. Therefore, this study provides a novel understanding of DNA damage and autophagy activity in NP cells and offers new therapeutic target for IDD.

## Materials and Methods

### Tissues Collection

Human NP tissues were collected from patients who underwent disc fusion surgery due to lumbar fracture or degenerative disc diseases. Informed consent was obtained from all patients who donated samples. The degree of IDD was assessed based on the Pfirrmann MRI-grade system (Liao et al., 2019b). Discs categorized as Pfirrmann grade I or II was considered as non-degenerative discs, while those categorized as grade III, IV, or V was degenerative discs. For the histological analysis, NP tissues were fixed in 4% formaldehyde and embedded in paraffin. Some samples were directly frozen in liquid nitrogen for protein and RNA extraction. All experimental protocols were approved by the Ethics Committee of Tongji Medical College, Huazhong University of Science and Technology.

### Cell Culture

NP cells were isolated using a previously described protocol (Wu et al., 2019). Briefly, NP tissues were cut into pieces and enzymatically digested in 0.2% type II collagenase for 4 h. After washed with PBS and centrifuged, these isolated cells were cultured in Dulbecco’s Modified Eagle Medium/Nutrient Mixture F-12 (DMEM/F-12) containing 15% fetal bovine serum in a 5% CO_2_ incubator. The culture medium was replaced twice per week. NP cells from the third passage were used for the subsequent experiments. For starvation culture, NP cells were cultured in Hanks Balanced Salt Solution (HBSS) for 4 h and then the autophagy levels were detected.

### Western Blot

Cells were collected and lysed in RIPA lysis buffer (Beyotime, China) with a protease inhibitor PMSF (Beyotime, China). The proteins were separated by sodium dodecyl sulfate polyacrylamide gel electrophoresis (SDS-PAGE) and transferred onto a PVDF membrane. The membranes were blocked with 5% milk for 1 h. The primary antibodies used were as follows: AIM2 (Proteintech, 1:2,000), ASC (Proteintech, 1:2,000), Caspase-1 (CST, 1:1,000), LC3 (Abcam, 1:1,000), GRASP55 (Proteintech, 1:1,000), GRASP65 (Proteintech, 1:1,000), CD63 (Proteintech, 1:500), TSG101 (Proteintech, 1:1,000), and Calnexin (Proteintech, 1:5,000). Horseradish peroxidase-conjugated secondary antibodies (Boster, China) were incubated with bands for 1 h, and bands were visualized and detected using the enhanced chemiluminescence system. The band intensity values of proteins were calculated using ImageJ 1.52a software (National Institutes of Health, United States).

### Enzyme-Linked Immunosorbent Assay

The cell supernatant was collected and centrifuged, then measured the level of IL-1β, IL-18, and AIM2 using the corresponding ELISA kit (Elabscience Biotechnology, China) according to the manufacturer’s protocol. The experiment was performed in triplicate.

### TUNEL Staining

TUNEL staining was used to assess cell apoptosis. Cells were fixed in 4% paraformaldehyde for 30 min and treated with 0.5% TritonX-100 for 10 min. After washed with PBS, cells were incubated with the TUNEL staining kit (Beyotime, China) according to the manufacturer’s instructions. Images were captured using a fluorescence microscope (Olympus, BX53, United States).

### Immunofluorescence Staining

NP cells were fixed with 4% paraformaldehyde and permeabilized with 0.2% Triton X-100 for 30 min. The cells in the slides were washed in PBS twice, and blocked with 2% goat serum for 1 h, and then incubated with primary antibodies. Nuclei were stained for 5 min with DAPI (Beyotime). Immunofluorescent images were captured using a fluorescence microscope (Olympus, BX53, United States) or a confocal microscope (Nikon A1R SI Confocal, Japan).

### Immunoprecipitation

Cell lysates were treated with 50 mM Tris-HCl, 150 mM NaCl, 1 mM EDTA, and 1% NP-40 with protease inhibitor cocktail (Beyotime). The sample (500 μg) was added with 10 μL of the following antibodies: AIM2 (Abcam), or LC3 (Abcam), and was incubated overnight at 4°C with magnetic beads. Then, the magnetic separated immunoprecipitates were conducted with Western blot assays.

### Transmission Electron Microscopy

NP cells were fixed in 2.5% glutaraldehyde overnight, post-fixed in 2% osmium tetroxide for 1 h and stained with 2% uranyl acetate for 1 h. After dehydration in an ascending series of acetone, the samples were embedded into Araldite. Samples were cut into ultrathin sections, and then stained with toluidine blue. Finally, sections were observed using a transmission electron microscope (TEM) (Tecnai G2 20 TWIN, FEI, United States). Randomized fields were captured and the autophagosomes in the field were counted.

### Knockdown Experiments

Knockdown of ATG5, AIM2, or GRASP55 in NP cells was achieved by transfection with small interfering RNA (siRNA). Target siRNA and scrambled siRNA (si-scr) were synthesized by RiboBio company (Guangzhou, China): ATG5-siRNA sequence 5′-GCUAUAUCAGGAUGAGAUATT-3′, AIM2-siRNA sequence 5′-GUCCCGCUGAACAUUAUCATT-3′, GRASP55-siRNA sequence 5′-GGUGGAAUCAAAUUCUC CUTT-3′. NP cells were transfected with Lipofectamine 2000 (Invitrogen) according to the manufacturer’s protocol. Transgenic efficacy in NP cells was detected using quantitative real-time polymerase chain reaction at 48 h after transfection.

### Quantitative Real-Time Polymerase Chain Reaction

RNA extracted with Trizol reagent (Invitrogen) from NP cells, was reverse-transcribed and amplified by Quantitative real-time polymerase chain reaction (qRT-PCR) according to the standard protocols. The qRT-PCR was performed to quantify mRNA expression levels. The primer sequences were listed below: Homo ATG5, forward 5′-AAAGATGTGCTT CGAGATGTGTGGT-3′, reverse 5′-GCAAATAGTATGGTTC TGCTTCCCT-3′; Homo AIM2, forward 5′-CAGAAGGT AACAGAAAAGAAGA-3′, reverse 5′-ACAGTGTGAAGAATG TAAGTC-3′; Homo GRASP55, forward 5′-CTGCGAGAGA CCTCAGTCACACCAA-3′, reverse 5′-ACCTCCAGCACAT GCCAAACATTTT-3′; Homo GAPDH, forward 5′-TCAAGAA GGTGGTGAAGCAGG-3′, reverse 5′-TCAAAGGTGGAGGAG TGGGT-3′. GAPDH was used for normalization. All data were tested in triplicate.

### Animal Experiments

Animal experiments were performed following protocols approved by the Animal Experimentation Committee of Huazhong University of Science and Technology. Sprague-Dawley (SD, male, 3 months) rats were purchased from the Experimental Animal Center of Tongji Medical College, Huazhong University of Science and Technology. A model of IDD was established by needle puncture (Chen et al., 2016; Liao et al., 2019b; Zheng et al., 2019). After the rats were anesthetized with 2% (w/v) pentobarbital (40 mg/kg), the IVD of rats (Co 8/9) was punctured with a 20-gauge needle from the dorsal side. Some rats remained intact as the control group. These rats were randomly divided into three groups and injected with: PBS (2 μL), *in vivo* AIM2-siRNA (2 μL, 100 μM) or scrambled siRNA (2 μL, 100 μM) using a 33-gauge needle (Hamilton, Benade, Switzerland). The injection procedure was repeated weekly for 2 months.

### Histologic Analysis

Animals were euthanized and the discs were harvested. The specimens were decalcified and fixed in formaldehyde, dehydrated and embedded in paraffin. The slides of each disc were stained with hematoxylin-eosin (HE) staining, safranin O (S-O) staining, and Sirius red (S-R) staining. The histological grades were evaluated based on histological staining as previously described (Liao et al., 2019a). For immunochemistry, the specimen sections were deparaffinized and rehydrated, and then microwaved in sodium citrate for 15 min. Next, 3% hydrogen peroxide was used to block endogenous peroxidase activity for 10 min, and 5% BSA was used to block non-specific binding sites for 30 min. The sections were then incubated with primary antibodies overnight at 4°C. Finally, the sections were incubated with a secondary antibody and counterstained with hematoxylin.

### Statistical Analysis

Data are presented as mean ± standard deviation (SD). Student’s *t*-test and one-way or two-way analysis of variance (ANOVA) with Tukey’s *post hoc* test were used to assess the changes in the effects for groups. Statistical significance was set at *P* < 0.05 (^∗^*P* or ^#^*P* < 0.05; ^∗∗^*P* or ^##^*P* < 0.01; ^∗∗∗^*P* or ^###^*P* < 0.001; *P* > 0.05, ns, no significant difference) and calculated using GraphPad Prism 8 software (La Jolla, CA, United States).

## Results

### Expression of AIM2 and DNA Damage Marker During Intervertebral Disc Degeneration

**FIGURE 1:**
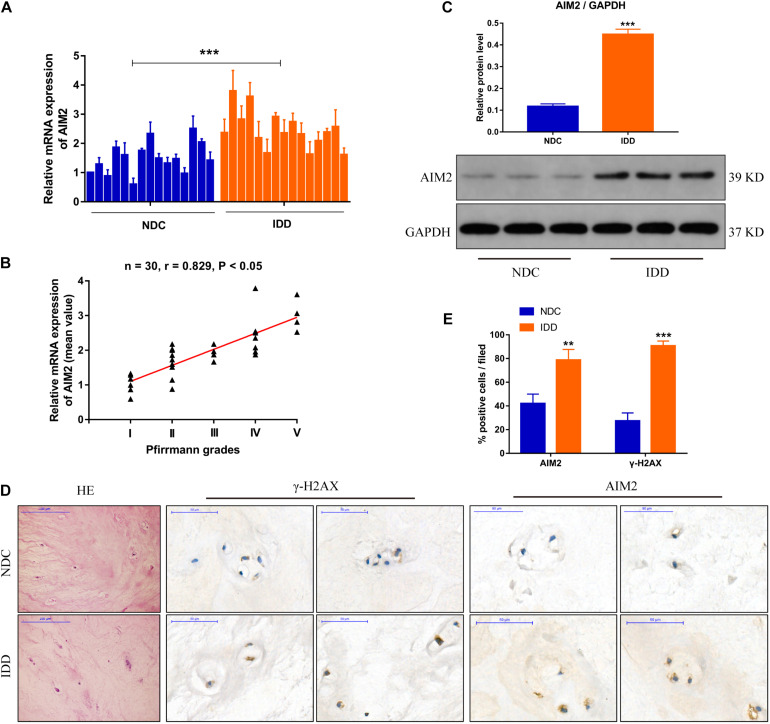
AIM2 expression in human intervertebral disc tissues. **(A)** AIM2 mRNA level measured by qRT-PCR in non-degenerative NP tissues (NDC) and degenerative NP tissues (IDD), *n* = 15. **(B)** Correlation analysis between the mean AIM2 mRNA level and Pfirrmann grades of NDC and IDD tissues. **(C)** Represent western blot image and quantification of AIM2 protein expression in human NDC and IDD tissues. GAPDH was used as an internal control. **(D)** HE staining and immunochemistry staining of γ-H2AX and AIM2 in human NDC and IDD tissues. **(E)** Quantification rate of AIM2 and γ-H2AX positive cells. Data were presented as the means ± SD, *n* = 3. **P < 0.01, ***P < 0.001 vs. NDC group.

We measured the transcriptional level of AIM2 in non-degenerative and degenerative NP tissues (Figure 1A). The correlation analysis between AIM2 level and disc degenerative degree indicated the increased AIM2 expression in IDD tissues (Figure 1B). We also assessed the protein levels of AIM2 in NP tissues (Figure 1C). The expression of AIM2 and γ-H2AX, an identified marker of DNA damage that accumulates in cells containing DNA damage (Lammert et al., 2020) was measured in non-degenerative and degenerative tissues via immunochemistry (Figure 1D). Higher levels of AIM2 and γ-H2AX were detected in the IDD tissues compared with the non-degenerative tissues (Figure 1E). These results indicated that expression level of AIM2 and degree of cellular DNA damage were elevated during IDD.

### AIM2 Inflammasome Contributes to Cellular DNA Damage in H_2_O_2_-Induced NP Cells

**FIGURE 2:**
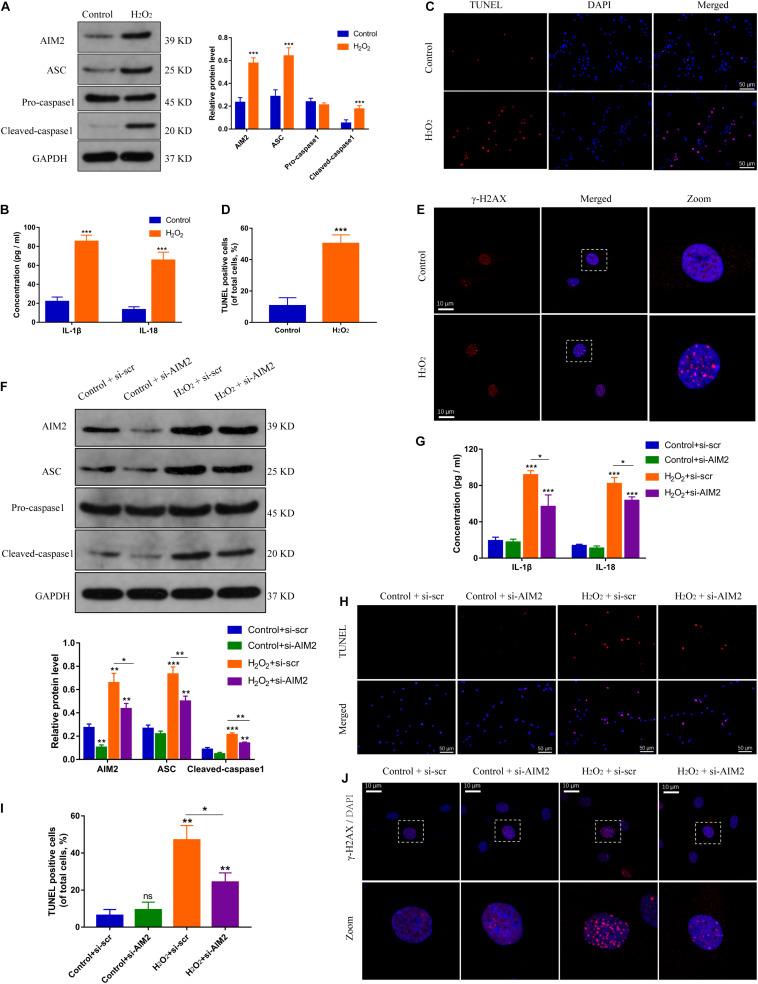
Silencing of AIM2 ameliorates DNA damage in NP cells *in vitro*. **(A)** Protein levels and quantification of AIM2, ASC, pro-caspase-1, and cleaved caspase-1 in H_2_O_2_-treated (200 μM, 8 h) NP cells. NP cells in the control group were treated with equivalent solvent. **(B)** Extracellular level of IL-1β and IL-18 measured by ELISA. **(C,D)** TUNEL analysis of H_2_O_2_-treated NP cells **(C)** and corresponding quantification of cell apoptosis **(D)**. **(E)** Immunostaining of γ-H2AX in H_2_O_2_-treated NP cells. Data were presented as the means ± SD. *P < 0.05, **P < 0.01, ***P < 0.001 vs. control group. **(F)** Protein levels and quantification of AIM2, ASC, pro-caspase-1, and cleaved caspase-1 in H_2_O_2_-treated NP cells co-treated with si-AIM2 or si-scr. **(G)** Extracellular level of IL-1β and IL-18 measured by ELISA. **(H,I)** Represent images of TUNEL analysis **(H)** and evaluation of cell apoptotic rates **(I)**. **(J)** Immunostaining of γ-H2AX in H_2_O_2_-treated NP cells co-treated with si-AIM2 or si-scr. *P < 0.05, **P < 0.01, ***P < 0.001 vs. control-si-scr group, *n* = 3.

Hydrogen peroxide (H_2_O_2_) could initiate a DNA damage response (Benkafadar et al., 2019), and was used to create a DNA damage model of NP cells. The expression levels of AIM2, the apoptosis associated speck-like protein containing a CARD (ASC), and cleaved caspase-1 were increased significantly in H_2_O_2_-treated NP cells (Figure 2A). Besides, the secretion of inflammatory cytokines IL-1β and IL-18 was also increased (Figure 2B). TUNEL analysis revealed an increased apoptotic rate of NP cells in the H_2_O_2_ group (Figures 2C,D). Immunostaining of γ-H2AX showed an accumulation in the cell nuclei (Figure 2E). Moreover, siRNA transfection was used to decrease the AIM2 expression, resulting in decreased level of ASC and cleaved caspase-1, and decreased secretion of IL-1β and IL-18 (Figures 2F,G). The knockdown of AIM2 inhibited NP cell apoptosis in the H_2_O_2_ group (Figures 2H,I). Besides, immunostaining of γ-H2AX revealed that H_2_O_2_-induced cellular DNA damage was ameliorated in the AIM2 knockdown group (Figure 2J). These results demonstrated that AIM2 plays a role in cellular DNA damage and AIM2 knockdown attenuates H_2_O_2_-induced DNA damage *in vitro.*

### Autophagy Regulates AIM2 Inflammasome Activation and Promotes AIM2 Secretion

**FIGURE 3:**
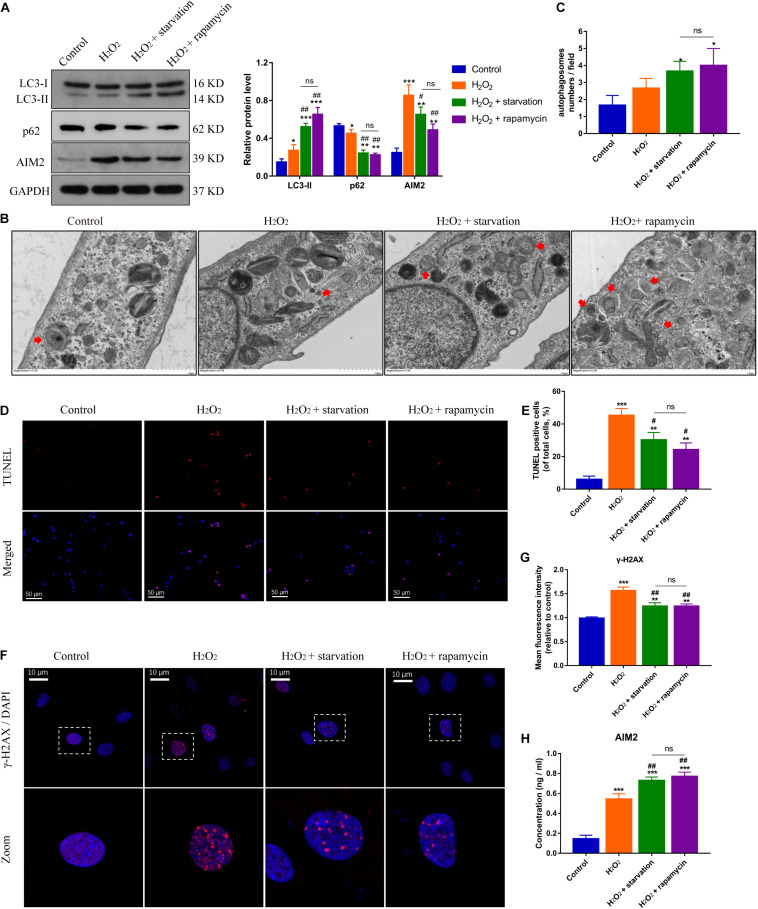
Induction of autophagy reduces DNA damage and NP cell apoptosis, and facilitates AIM2 secretion. **(A)** Protein levels and quantification of AIM2, LC3 and p62 in H_2_O_2_-treated NP cells pre-treated with starvation (8 h) or rapamycin (10 μM, 8 h). **(B,C)** TEM images of NP cells indicated the number and morphology of autophagosomes (red arrows), and quantification of autophagosomes numbers **(C)**. **(D,E)** TUNEL images of NP cells in different groups **(D)** and corresponding quantification of cell apoptosis **(E)**. **(F,G)** Immunostaining of γ-H2AX **(F)** and mean fluorescence intensity of NP cells in different groups **(G)**. **(H)** Secretion of AIM2 measured by ELISA analysis. Data were presented as the means ± SD, *n* = 3. *P < 0.05, **P < 0.01, ***P < 0.001 vs. control group; ^#^*P* < 0.05, ^##^*P* < 0.01 vs. H_2_O_2_ group; ns, *P* > 0.05, no significant difference.

Autophagy is closely related with inflammation response and cell apoptosis (Li et al., 2018). Here, we investigated the role of autophagy in AIM2 inflammasome activation. Starvation or rapamycin treatment induced autophagy activation and decreased the expression level of AIM2 in NP cells (Figure 3A). The morphology and number of autophagosomes in H_2_O_2_-treated NP cells was shown in transmission electron microscope (TEM) images (Figures 3B,C). Starvation or rapamycin treatment led to a decreased apoptotic rate (Figures 3D,E). Besides, γ-H2AX staining revealed that autophagy induction ameliorated H_2_O_2_-induced DNA damage (Figures 3F,G). Interestingly, the extracellular AIM2 levels increased upon autophagy activation (Figure 3H).

**FIGURE 4:**
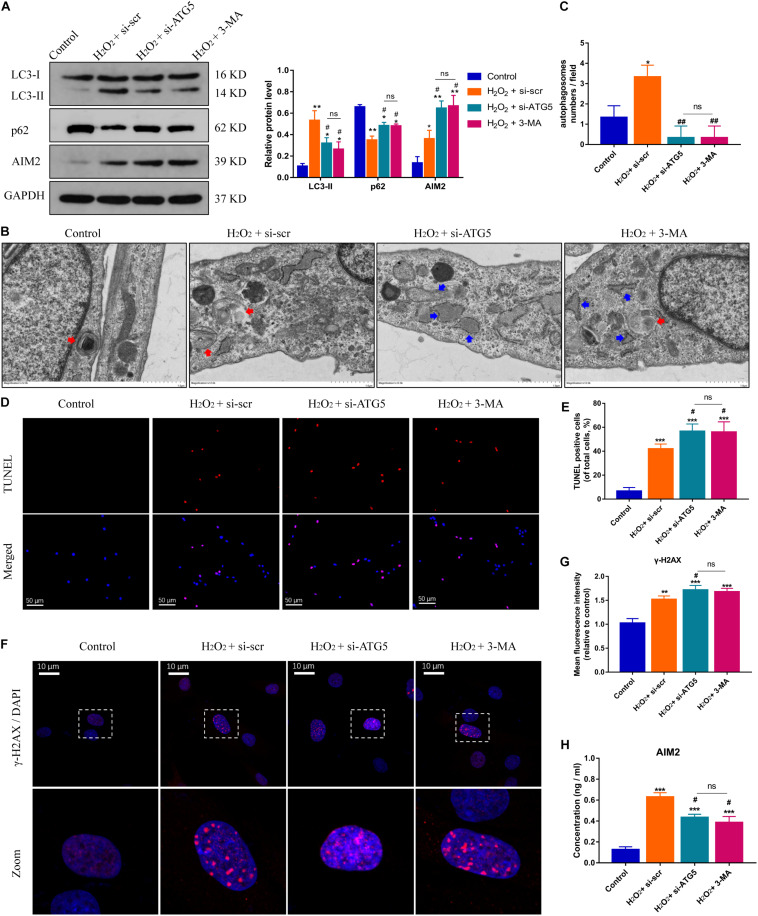
Inhibition of autophagy aggravates DNA damage and NP cell apoptosis, and decreases AIM2 secretion. **(A)** Protein levels and quantification of AIM2, LC3, and p62 in H_2_O_2_-treated NP cells co-treated with si-scr, si-ATG5, or 3-MA (20 mM, 8 h). **(B,C)** TEM images of NP cells indicated the number of autophagosomes (red arrows) and irregular intracellular vesicles (blue arrows), and quantification of autophagosomes numbers **(C)**. **(D,E)** TUNEL images of NP cells in different groups **(D)** and corresponding quantification of cell apoptosis **(E)**. **(F,G)** Immunostaining of γ-H2AX **(F)** and mean fluorescence intensity of NP cells in different groups **(G)**. **(H)** Extracellular levels of AIM2 measured by ELISA analysis. Data were presented as the means ± SD, *n* = 3. *P < 0.05, **P < 0.01, ***P < 0.001 vs. control group; ^#^*P* < 0.05, ^##^*P* < 0.01 vs. H_2_O_2_-si-scr group; ns, *P* > 0.05, no significant difference.

On the other hand, the protein levels of AIM2 were increased significantly in NP cells treated with si-ATG5 or 3-MA (an autophagy inhibitor) (Figure 4A). More irregular vesicles and fewer autophagosomes were found in the si-ATG5 or 3-MA group than in the control group (Figures 4B,C). Inhibition of autophagy also promoted H_2_O_2_-induced NP cell apoptosis (Figures 4D,E). Immunofluorescence analysis revealed that autophagy inhibition facilitated the accumulation of γ-H2AX in the cell nuclei (Figures 4F,G). Accordingly, we found the extracellular level of AIM2 decreased in the si-ATG5 or 3-MA group (Figure 4H). These results indicated that autophagy regulates AIM2 inflammasome activation and plays a role in the secretion of AIM2.

### AIM2 Secretion Is Unconventional Pathway and Independent of Extracellular Vesicles

**FIGURE 5:**
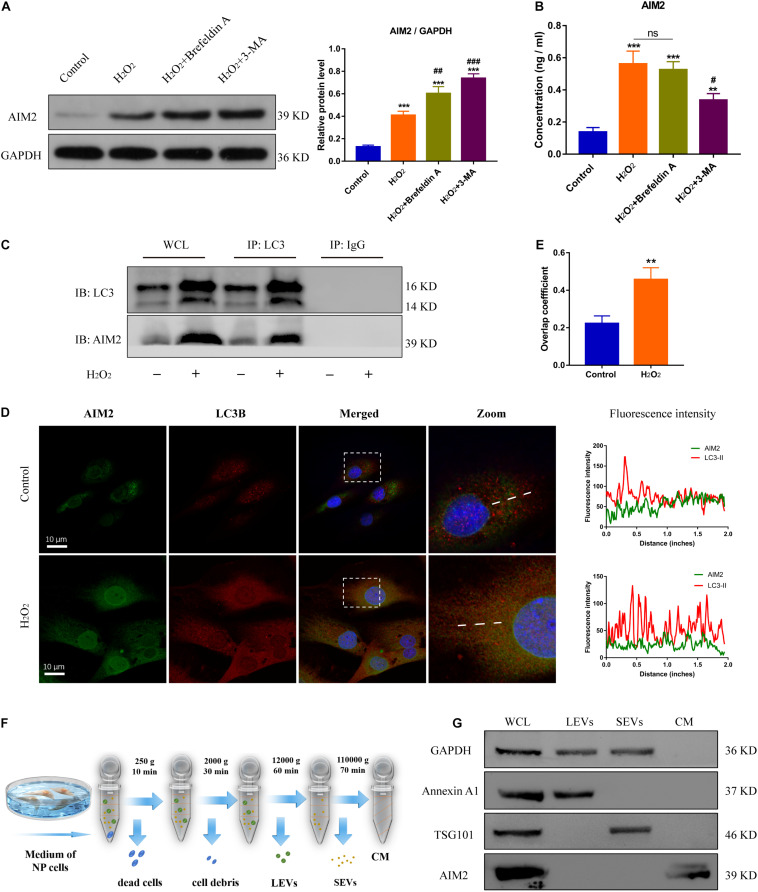
Autophagic secretory of AIM2 is independent of extracellular vesicles. **(A)** Protein levels and quantification of AIM2 in H_2_O_2_-treated NP cells co-treated with Brefeldin A (1 μg/ml, 8 h) or 3-MA. **(B)** Secretion of AIM2 measured by ELISA analysis in different groups. **(C)** Immunoprecipitation for LC3 was conducted to detect the integration of AIM2. IgG was as a negative control. WCL, whole cell lysate. **(D)** Confocal images of AIM2 (green) and LC3B (red) and fluorescence intensity results. **(E)** Overlap coefficient based on immunofluorescence images showed the colocalization between AIM2 and LC3B. Data were presented as the means ± SD, *n* = 3. **P < 0.01, ***P < 0.001 vs. control group; ^#^*P* < 0.05, ^##^*P* < 0.01, ^###^*P* < 0.001 vs. H_2_O_2_ group. **(F)** Workflow showed the isolation protocols and definition of extracellular vesicle fractions. LEVs, large extracellular vesicles; SEVs, small extracellular vesicles; CM, culture medium without extracellular vesicles. **(G)** Western blot analysis of extracellular vesicle markers and AIM2 in the different fractions. Annexin A1, a marker of LEVs; TSG101, a marker of SEVs; WCL, whole cell lysate.

To further confirm the role of autophagy in AIM2 secretion, NP cells were treated with 3-MA or Brefeldin A (an ER-Golgi transport inhibitor). The protein level of cellular AIM2 was increased in the 3-MA group (Figure 5A). Secretion of AIM2 was decreased in the 3-MA group while it did not have a significant change in the Brefeldin A group, indicating that AIM2 was secreted via an unconventional pathway (Figure 5B). Immunoprecipitation analysis of autophagic protein LC3 showed the integration between cellular AIM2 and LC3 (Figure 5C). Besides, we observed an overlap between LC3 positive puncta and AIM2 in H_2_O_2_-treated NP cells (Figures 5D,E). To further validate the secretory pathway of AIM2, we separated different secretome fractions from the NP cell culture medium, including large and small extracellular vesicles (Figure 5F). Western blot analysis showed that AIM2 was not detected in the extracellular vesicle fractions (Figure 5G). These results revealed that AIM2 secretion depends on the autophagy-based unconventional secretory pathway and its release is independent of extracellular vesicles.

### GRASP55 Mediates AIM2 Unconventional Secretion by Modulating Autophagy Activity

**FIGURE 6:**
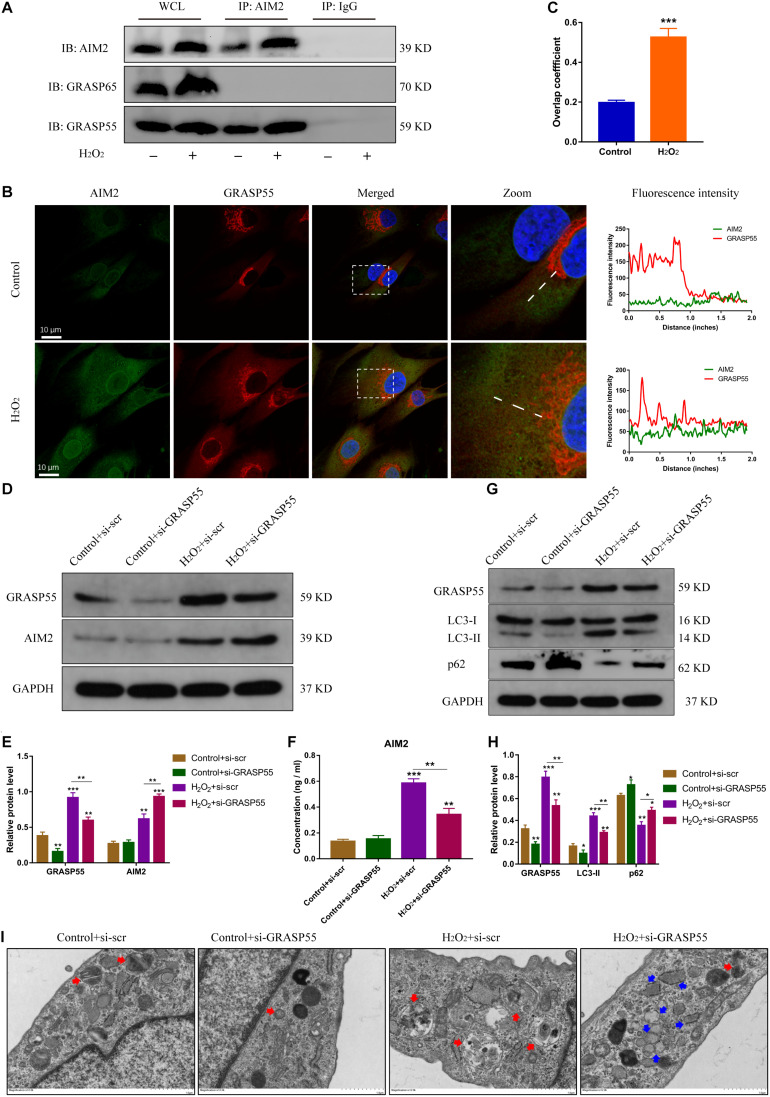
GRASP55 regulates AIM2 unconventional secretion and autophagy activity. **(A)** Immunoprecipitation for AIM2 was conducted to detect the integration of GRASP55 or GRASP65. **(B,C)** Confocal images of AIM2 (green) and GRASP55 (red) **(B)** and overlap coefficient **(C)** in H_2_O_2_-treated NP cells. **(D)** Knockdown of GRASP55 in NP cells and corresponding western blot images of GRASP55 and AIM2. **(E)** Quantification of AIM2 and GRASP55 in H_2_O_2_-treated NP cells with si-GRASP55 or si-scr. **(F)** Extracellular levels of AIM2 measured by ELISA analysis. **(G,H)** Protein levels **(G)** and quantification **(H)** of GRASP55, LC3 and p62 in H_2_O_2_-treated NP cells with si-GRASP55 or si-scr. Data were presented as the means ± SD, *n* = 3. *P < 0.05, **P < 0.01, ***P < 0.001 vs. control group. **(I)** TEM images of NP cells showed the autophagosomes (red arrows) and other irregular intracellular vesicles (blue arrows).

Several studies have reported the critical role of Golgi re-assembly and stacking proteins in the autophagic secretory pathway, including GRASP55 and GRASP65 (Dupont et al., 2011; Nuchel et al., 2018). We then investigated whether GRASP55 or GRASP65 were involved in AIM2 secretion. The immunoprecipitation of AIM2 detected the integration with GRASP55 and not GRASP65 (Figure 6A). Besides, we observed that AIM2 colocalized with GRASP55 and the overlap coefficient was increased in H_2_O_2_-treated NP cells (Figures 6B,C). Knockdown of GRASP55 promoted the accumulation of cytoplasmic AIM2 (Figures 6D,E). The extracellular level of AIM2 was decreased significantly in the si-GRASP55 group (Figure 6F). Moreover, the level of LC3-II was reduced in the si-GRASP55 group, indicating a decreased autophagic activity (Figures 6G,H). TEM images revealed several irregular vesicles and few autophagosomes in the si-GRASP55 group (Figure 6I). These results demonstrated that GRASP55 is indispensable for AIM2 secretion and regulates the secretory pathway via modulating autophagy activity.

### Knockdown of AIM2 Ameliorates Rat Disc Degeneration Progression *in vivo*

**FIGURE 7:**
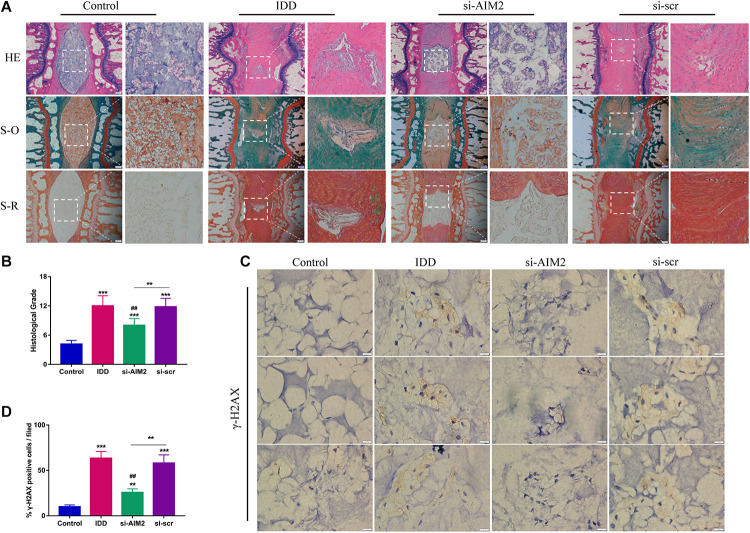
Inhibition of AIM2 retards IDD progression *in vivo*. **(A)** Histological staining, including hematoxylin-eosin (HE), safranin O (S-O), and Sirius red (S-R) staining, showed the morphology of rat disc tissues. The IDD, si-AIM2, and si-scr group were conducted with needle puncture. **(B)** Histological grades to evaluate the degree of disc degeneration in different group. **(C,D)** Immunochemistry results of γ-H2AX in rat dis tissues **(C)** and quantification of cγ-H2AX-positive cell rate **(D)**. Data were presented as the means ± SD, *n* = 6. **P < 0.01, ***P < 0.001 vs. control group; ^##^*P* < 0.01 vs. IDD group.

To further investigate the role of AIM2 in IDD, we constructed a rat disc model of IDD. Histological results showed that the nucleus pulposus was nearly diminished in the IDD and the si-scr group (Figure 7A). Besides, increased proteoglycans and fewer fibrosus tissues were detected in the si-AIM2 group than in the IDD group via safranin O and Sirius red staining, indicating a less degenerative profile (Figure 7A). Histological grades based on histological staining revealed that knockdown of AIM2 *in vivo* delays the progression of IDD (Figure 7B). Immunochemistry analysis showed that the rate of γ-H2AX positive staining cells was significantly decreased in the si-AIM2 group compared to the IDD group (Figures 7C,D). These results indicated that knockdown of AIM2 ameliorates cellular DNA damage and retards the progression of IDD *in vivo*.

## Discussion

**FIGURE 8:**
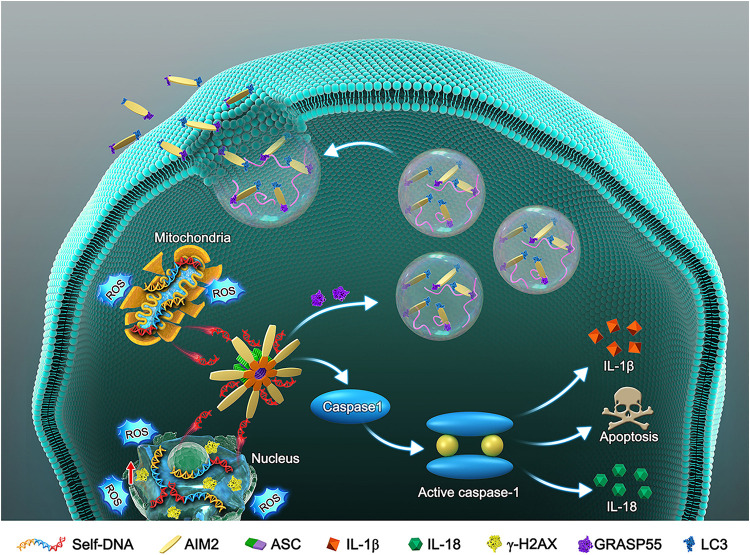
Schematic model illustrates the autophagic secretory pathway of AIM2 in NP cells. Oxidative stress-induced DNA damage activates the AIM2 inflammasome and elicits the downstream inflammation cascade. During this process, autophagy activation ameliorates DNA damage via an unconventional secretion of AIM2. AIM2, Absent in melanoma 2; ASC, Apoptosis-associated speck-like protein containing a CARD; NP, Nucleus pulposus; ROS, Reactive oxygen species; IL-1β, Interleukin-1b; IL-18, Interleukin 18; GRASP55, Golgi re-assembly and stacking protein 55.

Cellular DNA damage and inflammasome activation contribute greatly to the progression of IDD (Nasto et al., 2014; Cazzanelli and Wuertz-Kozak, 2020). The AIM2 inflammasome is a DNA damage sensor that activates the cleavage of caspase-1 and the release of inflammatory cytokines, resulting in cell apoptosis or pyroptosis (Sharma et al., 2019). Here, we evaluated the relationship between AIM2 inflammasome expression and IDD in human disc tissue samples. AIM2 knockdown significantly ameliorated cellular DNA damage and cell death induced by H_2_O_2_ *in vitro*. Especially, we investigated the secretory mechanism of AIM2 in adaption to cellular DNA damage (Figure 8). AIM2 was found to be colocalized with LC3 upon autophagy activation and cooperated with GRASP55, which assisted the extracellularly release of AIM2. In the *in vivo* experiments, AIM2 knockdown delayed disc degeneration, confirming the detrimental role of AIM2 in the progression of IDD.

The major components of intervertebral disc (IVD) are water and ECM proteins, such as proteoglycans and collagens (Tendulkar et al., 2019). NP cells are distributed in the central of IVD and surrounded by ECM components. Several studies have indicated that damaged or aging NP cells accelerates the degradation of ECM components, and diminishing of resident cells decreased the synthesis of proteoglycans and collagens (Dimozi et al., 2015; Wang et al., 2016). Oxidative stress and DNA damage contribute significantly to NP cell injury, which plays a vital role in the progression of IDD (Nasto et al., 2014; Feng et al., 2017). Oxidative stress causes irreversible DNA damage, which is closely related to mitochondrial dysfunction and cell senescence (Hyttinen et al., 2017). In addition, DNA damage can induce the activation of the AIM2 inflammasome, resulting in a pro-inflammatory phenotype, or programmed cell death, such as apoptosis and pyroptosis (Benkafadar et al., 2019; Sharma et al., 2019). Both disordered inflammation and programmed cell death are critical culprits in IDD. H_2_O_2_-induced DNA damage activates the AIM2 inflammasome and elicits inflammatory cascades, ultimately promoting the degeneration of NP cells. Knockdown of AIM2 significantly reduces the production of inflammatory cytokines and ameliorates NP cell death.

Autophagy, especially macroautophagy, has been implicated in various cellular physiological and pathological processes (Mizushima et al., 2010). Several studies have indicated that autophagy activation in NP cells attenuates cell apoptosis (Li et al., 2018; Tang et al., 2019); however, the effects of autophagy are complicated, as higher or lower levels may induce a detrimental effect on different types of cells. Although well understood as a degradative way, autophagy also promotes the transport and secretion of specific substrates (Jiang et al., 2013). Some proteins are secreted through a secretory autophagy route, which is mediated by secretory autophagosome trafficking (Ponpuak et al., 2015). A previous study revealed that extracellular delivery of IL-1β depends on the autophagy-based unconventional secretory pathway rather than the typical endoplasmic reticulum-Golgi pathway (Dupont et al., 2011). Some cytokines, immune mediators, and ECM molecules also rely on the autophagy-based unconventional secretory pathway (Thorburn et al., 2008; Endo et al., 2017; Nuchel et al., 2018). Unlike degrative autophagy aimed at protein degradation and recycling, secretory autophagy could eliminate intracellular harmful proteins or aggregates by selective recruitment and trafficking (Urano et al., 2018). A research found that level of extracellular AIM2 was increased in a disease model, while the secretory mechanism of AIM2 was not investigated yet (Yuan et al., 2020). In our study, it demonstrated that AIM2 secretion was closely related with secretory autophagy. We found that intracellular level of AIM2 decreased while extracellular AIM2 increased upon autophagy activation. The autophagy-based unconventional secretory pathway may serve as a protective mechanism for cells adapting AIM2-activatied inflammatory cascades. However, it was also reasonable to assume the degradative mechanism of AIM2 upon autophagy activation, which may be another adjustive mechanism of NP cells.

Autophagy-dependent secretion is a kind of unconventional protein secretion (UPS). Diverse types of intracellular membrane structures are involved in UPS, and are responsible for cargo recruitment and transportation, including autophagosomes, late endosomes, and lysosomes (New and Thomas, 2019). Golgi Re-Assembly and Stacking Proteins (GRASPs) which have been implicated in the unconventional secretion of IL-1β and TGF-β, are important participants in secretory autophagy, mediating the protein trafficking (Dupont et al., 2011; Giuliani et al., 2011; Nuchel et al., 2018). Especially, GRASPs are required for stress-induced unconventional secretion, such as under conditions of inflammation, starvation, or mechanical stress (Duran et al., 2010; Giuliani et al., 2011; Kim et al., 2016). Before the fusion with plasma membrane, autophagosomes loaded with specific proteins are decorated by some Soluble NSF Attachment Protein Receptors (SNARE). Mediated by a SNARE complex, the secretory autophagosomes mix with the plasma membrane and facilitate the protein secretion (Cruz-Garcia et al., 2018; New and Thomas, 2019; Wang et al., 2020). Our results showed that AIM2 colocalized with LC3-positive vesicles and the secretion of AIM2 was increased upon autophagy activation. The GRASP protein was also colocalized with AIM2, and was required for AIM2 secretion. Besides autophagosomes, exosomes and other extracellular vesicles were also possibly involved in AIM2 secretion. However, our results found that AIM2 was not detected in the extracellular vesicles fraction. The activation of autophagy could reduce intracellular AIM2 levels by promoting secretory autophagy, and this discovery may serve as a potential therapeutic target for inflammation-related IDD.

Our research innovatively illustrates the role of the AIM2 inflammasome in IDD, and reveals the effect of AIM2 inflammasome activation on the progression of IDD. Oxidative stress-induced DNA damage activates the AIM2 inflammasome and elicits the inflammatory cascades. Our study also demonstrates that autophagy protects against the activation of the AIM2 inflammasome via an unconventional secretory pathway. However, there still are several limitations in our research. Firstly, the knockdown of AIM2 was realized by siRNA while a gene knock-out technique may provide more reliable results. Secondly, the regulatory mechanism of autophagy on AIM2 may not be restricted to secretory autophagy. The involved mechanisms may be diverse and multifaceted. Thirdly, in addition to GRASP proteins, many proteins and signaling pathways were participated in unconventional secretion, which may also play a role in this process. Hence, further researches on the role of autophagy in the AIM2 inflammasome activation are required, especially in the field of musculoskeletal degenerative diseases.

In conclusion, the present study represents the first demonstration of the role of AIM2 inflammasome in IDD. We investigated the detrimental role of AIM2 in mediating DNA damage-associated NP cell inflammation and apoptosis. During IDD progression, autophagy regulates the secretion of AIM2 and serves as a protective mechanism. Moreover, knockdown of AIM2 ameliorates cellular DNA damage and disc degeneration *in vitro* and *in vivo*. Thus, our study illustrates the relationship between the AIM2 inflammasome and IDD, which may provide a potential therapeutical target for IDD treatment.

## Data Availability Statement

The original contributions presented in the study are included in the article/supplementary material, further inquiries can be directed to the corresponding author/s.

## Ethics Statement

The studies involving human participants were reviewed and approved by the Ethics Committee of Tongji Medical College, Huazhong University of Science and Technology. The patients/participants provided their written informed consent to participate in this study. The animal study was reviewed and approved by the Animal Experimentation Committee of Huazhong University of Science and Technology.

## Author Contributions

CY and HY extensively reviewed this manuscript and provided funding. SL and ZL designed and conducted this study. All authors have reviewed and given approval to the final version of the manuscript.

## Conflict of Interest

The authors declare that the research was conducted in the absence of any commercial or financial relationships that could be construed as a potential conflict of interest.

## References

[B1] Benkafadar N. Francois F. Affortit C. Casas F. Ceccato J. C. Menardo J. (2019). ROS-Induced Activation of DNA damage responses drives senescence-like state in postmitotic cochlear cells: implication for hearing preservation. *Mol. Neurobiol.* 56 5950–5969. 10.1007/s12035-019-1493-6 30693443 PMC6614136

[B2] Cazzanelli P. Wuertz-Kozak K. (2020). MicroRNAs in intervertebral disc degeneration, apoptosis, inflammation, and mechanobiology. *Int. J. Mol. Sci.* 21:3601. 10.3390/ijms21103601 32443722 PMC7279351

[B3] Chen D. Xia D. Pan Z. Xu D. Zhou Y. Wu Y. (2016). Metformin protects against apoptosis and senescence in nucleus pulposus cells and ameliorates disc degeneration in vivo. *Cell Death Dis.* 7:e2441. 10.1038/cddis.2016.334 27787519 PMC5133996

[B4] Cruz-Garcia D. Malhotra V. Curwin A. J. (2018). Unconventional protein secretion triggered by nutrient starvation. *Semin. Cell Dev. Biol.* 83 22–28. 10.1016/j.semcdb.2018.02.021 29486236

[B5] Dimozi A. Mavrogonatou E. Sklirou A. Kletsas D. (2015). Oxidative stress inhibits the proliferation, induces premature senescence and promotes a catabolic phenotype in human nucleus pulposus intervertebral disc cells. *Eur. Cell Mater.* 30 89–102. 10.22203/ecm.v030a07 26337541

[B6] Dowdell J. Erwin M. Choma T. Vaccaro A. Iatridis J. Cho S. K. (2017). Intervertebral disk degeneration and repair. *Neurosurgery* 80 S46–S54. 28350945 10.1093/neuros/nyw078 PMC5585783

[B7] Dupont N. Jiang S. Pilli M. Ornatowski W. Bhattacharya D. Deretic V. (2011). Autophagy-based unconventional secretory pathway for extracellular delivery of IL-1β. *EMBO J.* 30 4701–4711. 10.1038/emboj.2011.398 22068051 PMC3243609

[B8] Duran J. M. Anjard C. Stefan C. Loomis W. F. Malhotra V. (2010). Unconventional secretion of Acb1 is mediated by autophagosomes. *J. Cell Biol.* 188 527–536. 10.1083/jcb.200911154 20156967 PMC2828925

[B9] Endo S. Nakata K. Ohuchida K. Takesue S. Nakayama H. Abe T. (2017). Autophagy is required for activation of pancreatic stellate cells, associated with pancreatic cancer progression and promotes growth of pancreatic tumors in mice. *Gastroenterology* 152 1492–1506. 10.1053/j.gastro.2017.01.010 28126348

[B10] Feng C. Yang M. Lan M. Liu C. Zhang Y. Huang B. (2017). ROS: crucial intermediators in the pathogenesis of intervertebral disc degeneration. *Oxid. Med. Cell. Longev.* 2017:5601593. 10.1155/2017/5601593 PMC5368368 28392887

[B11] Giuliani F. Grieve A. Rabouille C. (2011). Unconventional secretion: a stress on GRASP. *Curr. Opin. Cell Biol.* 23 498–504. 10.1016/j.ceb.2011.04.005 21571519

[B12] He X. F. Zeng Y. X. Li G. Feng Y. K. Wu C. Liang F. Y. (2020). Extracellular ASC exacerbated the recurrent ischemic stroke in an NLRP3-dependent manner. *J. Cereb. Blood Flow Metab.* 40 1048–1060. 10.1177/0271678x19856226 31216943 PMC7181081

[B13] Hyttinen J. M. T. Błasiak J. Niittykoski M. Kinnunen K. Kauppinen A. Salminen A. (2017). DNA damage response and autophagy in the degeneration of retinal pigment epithelial cells—Implications for age-related macular degeneration (AMD). *Ageing Res. Rev.* 36 64–77. 10.1016/j.arr.2017.03.006 28351686

[B14] Jiang S. Dupont N. Castillo E. F. Deretic V. (2013). Secretory versus degradative autophagy: unconventional secretion of inflammatory mediators. *J. Innate Immun.* 5 471–479. 10.1159/000346707 23445716 PMC3723810

[B15] Kim J. Noh S. H. Piao H. Kim D. H. Kim K. Cha J. S. (2016). Monomerization and ER relocalization of GRASP is a requisite for unconventional secretion of CFTR. *Traffic* 17 733–753. 10.1111/tra.12403 27062250

[B16] Kumari P. Russo A. J. Shivcharan S. Rathinam V. A. (2020). AIM2 in health and disease: inflammasome and beyond. *Immunol. Rev.* 297 83–95. 10.1111/imr.12903 32713036 PMC7668394

[B17] Lammert C. R. Frost E. L. Bellinger C. E. Bolte A. C. Mckee C. A. Hurt M. E. (2020). AIM2 inflammasome surveillance of DNA damage shapes neurodevelopment. *Nature* 580 647–652. 10.1038/s41586-020-2174-3 32350463 PMC7788527

[B18] Lan T. Shiyu H. Shen Z. Yan B. Chen J. (2020). New insights into the interplay between miRNA and autophagy in the ageing of intervertebral disc. *Ageing Res. Rev.* 65:101227. 10.1016/j.arr.2020.101227 33238206

[B19] Li S. Hua W. Wang K. Gao Y. Chen S. Liu W. (2018). Autophagy attenuates compression-induced apoptosis of human nucleus pulposus cells via MEK/ERK/NRF1/Atg7 signaling pathways during intervertebral disc degeneration. *Exp. Cell Res.* 370 87–97. 10.1016/j.yexcr.2018.06.012 29908161

[B20] Liao Z. Luo R. Li G. Song Y. Zhan S. Zhao K. (2019a). Exosomes from mesenchymal stem cells modulate endoplasmic reticulum stress to protect against nucleus pulposus cell death and ameliorate intervertebral disc degeneration in vivo. *Theranostics* 9 4084–4100. 10.7150/thno.33638 31281533 PMC6592170

[B21] Liao Z. Wu X. Song Y. Luo R. Yin H. Zhan S. (2019b). Angiopoietin-like protein 8 expression and association with extracellular matrix metabolism and inflammation during intervertebral disc degeneration. *J. Cell Mol. Med.* 23 5737–5750. 10.1111/jcmm.14488 31211513 PMC6653761

[B22] Lugrin J. Martinon F. (2018). The AIM2 inflammasome: sensor of pathogens and cellular perturbations. *Immunol. Rev.* 281 99–114. 10.1111/imr.12618 29247998

[B23] Madabhushi R. Pan L. Tsai L. H. (2014). DNA damage and its links to neurodegeneration. *Neuron* 83 266–282. 10.1016/j.neuron.2014.06.034 25033177 PMC5564444

[B24] Mizushima N. Komatsu M. (2011). Autophagy: renovation of cells and tissues. *Cell* 147 728–741. 10.1016/j.cell.2011.10.026 22078875

[B25] Mizushima N. Yoshimori T. Levine B. (2010). Methods in mammalian autophagy research. *Cell* 140 313–326. 10.1016/j.cell.2010.01.028 20144757 PMC2852113

[B26] Nasto L. A. Ngo K. Leme A. S. Robinson A. R. Dong Q. Roughley P. (2014). Investigating the role of DNA damage in tobacco smoking-induced spine degeneration. *Spine J.* 14 416–423. 10.1016/j.spinee.2013.08.034 24211096 PMC3944725

[B27] New J. Thomas S. M. (2019). Autophagy-dependent secretion: mechanism, factors secreted, and disease implications. *Autophagy* 15 1682–1693. 10.1080/15548627.2019.1596479 30894055 PMC6735501

[B28] Nuchel J. Ghatak S. Zuk A. V. Illerhaus A. Morgelin M. Schonborn K. (2018). TGFB1 is secreted through an unconventional pathway dependent on the autophagic machinery and cytoskeletal regulators. *Autophagy* 14 465–486. 10.1080/15548627.2017.1422850 29297744 PMC5915026

[B29] Ponpuak M. Mandell M. A. Kimura T. Chauhan S. Cleyrat C. Deretic V. (2015). Secretory autophagy. *Curr. Opin. Cell Biol.* 35 106–116. 25988755 10.1016/j.ceb.2015.04.016 PMC4529791

[B30] Risbud M. V. Shapiro I. M. (2014). Role of cytokines in intervertebral disc degeneration: pain and disc content. *Nat. Rev. Rheumatol.* 10 44–56. 10.1038/nrrheum.2013.160 24166242 PMC4151534

[B31] Sharma B. R. Karki R. Kanneganti T. D. (2019). Role of AIM2 inflammasome in inflammatory diseases, cancer and infection. *Eur. J. Immunol.* 49 1998–2011. 10.1002/eji.201848070 31372985 PMC7015662

[B32] Tang Z. Hu B. Zang F. Wang J. Zhang X. Chen H. (2019). Nrf2 drives oxidative stress-induced autophagy in nucleus pulposus cells via a Keap1/Nrf2/p62 feedback loop to protect intervertebral disc from degeneration. *Cell Death Dis.* 10:510. 10.1038/s41419-019-1701-3 PMC6602960 31263165

[B33] Tendulkar G. Chen T. Ehnert S. Kaps H. P. Nüssler A. K. (2019). Intervertebral disc nucleus repair: hype or hope? *Int. J. Mol. Sci.* 20:3622. 10.3390/ijms20153622 31344903 PMC6696292

[B34] Thorburn J. Horita H. Redzic J. Hansen K. Frankel A. E. Thorburn A. (2008). Autophagy regulates selective HMGB1 release in tumor cells that are destined to die. *Cell Death Differ.* 16 175–183. 10.1038/cdd.2008.143 18846108 PMC2605182

[B35] Urano Y. Mori C. Fuji A. Konno K. Yamamoto T. Yashirogi S. (2018). 6-Hydroxydopamine induces secretion of PARK7/DJ-1 via autophagy-based unconventional secretory pathway. *Autophagy* 14 1943–1958. 10.1080/15548627.2018.1493043 30112966 PMC6152502

[B36] Vergroesen P. P. Kingma I. Emanuel K. S. Hoogendoorn R. J. Welting T. J. Van Royen B. J. (2015). Mechanics and biology in intervertebral disc degeneration: a vicious circle. *Osteoarthritis Cartilage* 23 1057–1070. 10.1016/j.joca.2015.03.028 25827971

[B37] Wang F. Cai F. Shi R. Wang X. H. Wu X. T. (2016). Aging and age related stresses: a senescence mechanism of intervertebral disc degeneration. *Osteoarthritis Cartilage* 24 398–408. 10.1016/j.joca.2015.09.019 26455958

[B38] Wang Z. Zhou H. Zheng H. Zhou X. Shen G. Teng X. (2020). Autophagy-based unconventional secretion of HMGB1 by keratinocytes plays a pivotal role in psoriatic skin inflammation. *Autophagy* 17 529–552. 10.1080/15548627.2020.1725381 32019420 PMC8007160

[B39] Wu X. Liao Z. Wang K. Hua W. Liu X. Song Y. (2019). Targeting the IL-1beta/IL-1Ra pathways for the aggregation of human islet amyloid polypeptide in an ex vivo organ culture system of the intervertebral disc. *Exp. Mol. Med.* 51 1–16. 10.1038/s12276-019-0310-7 31554783 PMC6802624

[B40] Yuan B. Zhou X.-M. You Z.-Q. Xu W.-D. Fan J.-M. Chen S.-J. (2020). Inhibition of AIM2 inflammasome activation alleviates GSDMD-induced pyroptosis in early brain injury after subarachnoid haemorrhage. *Cell Death Dis.* 11:76. 10.1038/s41419-020-2248-z PMC6992766 32001670

[B41] Zhang S.-J. Yang W. Wang C. He W.-S. Deng H.-Y. Yan Y.-G. (2016). Autophagy: a double-edged sword in intervertebral disk degeneration. *Clin. Chim. Acta* 457 27–35. 10.1016/j.cca.2016.03.016 27018178

[B42] Zheng G. Pan Z. Zhan Y. Tang Q. Zheng F. Zhou Y. (2019). TFEB protects nucleus pulposus cells against apoptosis and senescence via restoring autophagic flux. *Osteoarthritis Cartilage* 27 347–357. 10.1016/j.joca.2018.10.011 30414849

